# 4D-CT strain analysis for biomechanical characterization of COPD in patients undergoing radiotherapy planning: an exploratory study

**DOI:** 10.3389/fmed.2025.1703045

**Published:** 2026-01-12

**Authors:** Qi Dai, Xiaoxiao Zhu, Hai Chen, Xun Wang, Bin Chen, Jingyun Shi

**Affiliations:** 1 School of Medicine, Tongji University, Shanghai, China; 2 Department of Radiology, Ningbo No.2 Hospital, Ningbo, China; 3 Department of Respiratory and Critical Care Medicine, Ningbo No.2 Hospital, Ningbo, China; 4 Department of Radiation Technology, Ningbo No.2 Hospital, Ningbo, China; 5 Department of Radiology, Shanghai Pulmonary Hospital, School of Medicine, Tongji University, Shanghai, China

**Keywords:** 4D-CT, chronic obstructive pulmonary disease, functional imaging, pulmonary biomechanics, radiotherapy planning, strain analysis

## Abstract

**Background:**

Chronic obstructive pulmonary disease (COPD) is characterized by progressive airflow limitation and altered pulmonary biomechanics. While conventional spirometry provides global functional information, it cannot assess regional heterogeneity. This exploratory study aimed to evaluate the diagnostic performance of four-dimensional computed tomography (4D-CT) strain analysis in differentiating normal lung function from COPD in a cohort of patients with small pulmonary lesions who underwent 4D-CT as part of radiotherapy planning, thereby establishing feasibility for potential future integration into treatment workflows.

**Methods:**

This single-center retrospective cross-sectional study included 46 patients with pulmonary lesions ≤ 3 cm who underwent 4D-CT localization scanning for radiotherapy planning between January 2021 and March 2024. Patients were stratified into normal lung function (*n* = 34; FEV_1_/FVC ≥ 0.7 and FEV_1_ ≥ 80% predicted) and COPD groups (*n* = 12; FEV_1_/FVC < 0.7) based on spirometric results according to GOLD criteria. Strain parameters—maximum principal strain (PSmax), mean principal strain (PSmean), and maximum displacement velocity (Speedmax)—were calculated using optical flow algorithms. Internal validation using bootstrap resampling (1,000 iterations) and multivariate analysis adjusting for tumor size were performed. All lung parenchyma, including tumor regions, was included in the strain analysis to reflect real-world clinical scenarios.

**Results:**

Despite the small COPD sample size (*n* = 12), the study achieved adequate statistical power (88.1%) due to large effect sizes. COPD patients exhibited significantly higher symptom prevalence and severely impaired pulmonary function (FEV_1_/FVC: 58.44 ± 8.78% vs. 82.31 ± 6.41%, *P* < 0.001, Cohen’s *d* = 3.373). Speedmax demonstrated statistically significant reduction in COPD patients (763.22 ± 85.22 vs. 1074.19 ± 319.68 mm/s, *P* < 0.001, *d* = 1.114), while PSmax and PSmean showed trends toward reduction. Speedmax demonstrated strong correlations with pulmonary function indices, particularly with FEV_1_/FVC (*r* = 0.533, *P* < 0.01), FEV_1_ (*r* = 0.445, *P* < 0.01), and MEF_50_ (*r* = 0.525, *P* < 0.01). Receiver operating characteristic analysis revealed good diagnostic performance for Speedmax (AUC = 0.886, optimism-corrected AUC = 0.889, sensitivity = 100.0%, specificity = 72.7%). Multivariate analysis confirmed that Speedmax remained a significant independent predictor of COPD status after adjusting for tumor size (AUC = 0.931). Bootstrap internal validation showed minimal optimism bias (-0.001), confirming model robustness.

**Conclusion:**

This exploratory study provides preliminary evidence that 4D-CT strain analysis, particularly Speedmax, shows promise for differentiating normal lung function from COPD in patients with small pulmonary lesions. However, given the small sample size and single-center design, diagnostic performance metrics should be interpreted with caution. External validation in larger, multi-center cohorts is required before clinical implementation.

## 1 Introduction

Chronic obstructive pulmonary disease (COPD) represents a major global health burden, affecting over 300 million people worldwide and ranking among the leading causes of morbidity and mortality (1). The 2023 Global Initiative for Chronic Obstructive Lung Disease (GOLD) guidelines define COPD as a heterogeneous lung condition characterized by chronic respiratory symptoms and persistent, usually progressive airflow obstruction (2). Disease burden is projected to increase substantially in the coming decades, particularly among aging populations (3).

Spirometry, specifically the forced expiratory volume in 1 s to forced vital capacity ratio (FEV_1_/FVC), remains the diagnostic gold standard for COPD (4). However, spirometry provides only global functional information and cannot capture the regional heterogeneity characteristic of COPD pathophysiology. Moreover, spirometry has limited sensitivity for early-stage disease detection and may be unfeasible in certain clinical scenarios, such as critically ill patients or those with contraindications to pulmonary function testing (5).

Computed tomography (CT) has emerged as a valuable tool for COPD phenotyping and disease monitoring (6). Quantitative CT analysis enables assessment of emphysema extent, airway remodeling, and small airway disease, providing insights beyond traditional functional measurements (7, 8). However, conventional CT provides only static anatomical information and cannot capture the dynamic aspects of respiratory mechanics. Four-dimensional CT (4D-CT) technology has opened new avenues for evaluating dynamic lung function by capturing temporal changes throughout the respiratory cycle (9). 4D-CT strain analysis represents an innovative approach to quantifying regional lung biomechanics (10). Originating from materials science for tissue deformation quantification, strain analysis has been successfully applied to cardiac imaging before extending to pulmonary applications (11). By analyzing lung tissue deformation during respiration, strain analysis provides quantitative measures of regional lung mechanics, elasticity, and motion characteristics (12).

COPD pathophysiology involves progressive alveolar wall destruction, elastic recoil loss, and airway remodeling, all fundamentally altering lung tissue mechanical properties (13). Strain analysis may capture these biomechanical changes before they become apparent on conventional imaging or spirometry, potentially enabling earlier detection and more precise disease characterization (14). This capability is particularly relevant in the context of precision medicine approaches, where individualized assessment of lung function could guide therapeutic decision-making.

In radiation oncology, accurate lung function assessment is crucial for treatment planning and risk stratification (15). Lung cancer patients commonly present with coexisting COPD, making pulmonary reserve assessment essential for optimizing treatment approaches and predicting treatment-related complications such as radiation pneumonitis (16). The prevalence of COPD in lung cancer patients ranges from 40 to 70%, significantly higher than in the general population, largely due to shared risk factors, particularly tobacco smoking (17). 4D-CT utilization for radiotherapy planning provides a unique opportunity to extract additional functional information without increased radiation exposure or patient burden.

The integration of functional imaging with radiotherapy planning has gained considerable attention in recent years, with the concept of functional lung avoidance becoming increasingly important for reducing treatment-related toxicity (18). By identifying and preserving regions of high functional lung tissue, radiation treatment plans can potentially minimize pulmonary complications while maintaining oncological efficacy. 4D-CT strain analysis could provide the regional functional information necessary to implement such strategies effectively.

This exploratory study systematically evaluated the diagnostic performance of 4D-CT strain analysis in differentiating normal lung function from COPD in patients with small pulmonary lesions (≤ 3 cm) undergoing radiotherapy planning. This study establishes the diagnostic potential of strain analysis within the radiotherapy patient population, providing foundational evidence for potential future applications in functional lung avoidance treatment planning, without actual integration into treatment planning systems at this stage. By focusing on this specific population and implementing rigorous quality control measures, we sought to establish strain analysis feasibility and clinical utility while minimizing confounding factors associated with large pulmonary lesions that could significantly alter regional biomechanics.

## 2 Materials and methods

### 2.1 Study design and ethics

This single-center retrospective cross-sectional study was conducted through systematic review of radiation oncology department patient records at Ningbo No.2 Hospital. Clinical data and imaging studies were retrospectively collected from hospital information system (HIS) and radiology information system (RIS) databases for patients undergoing 4D-CT localization scanning between January 2021 and March 2024. The institutional review board approved the study protocol (Ethics approval: YJ-NBEY-KY-2023-107-01), conducted in accordance with the Declaration of Helsinki. Given the retrospective design with no alteration to standard patient care, informed consent requirements were waived.

### 2.2 Study population

**FIGURE 1:**
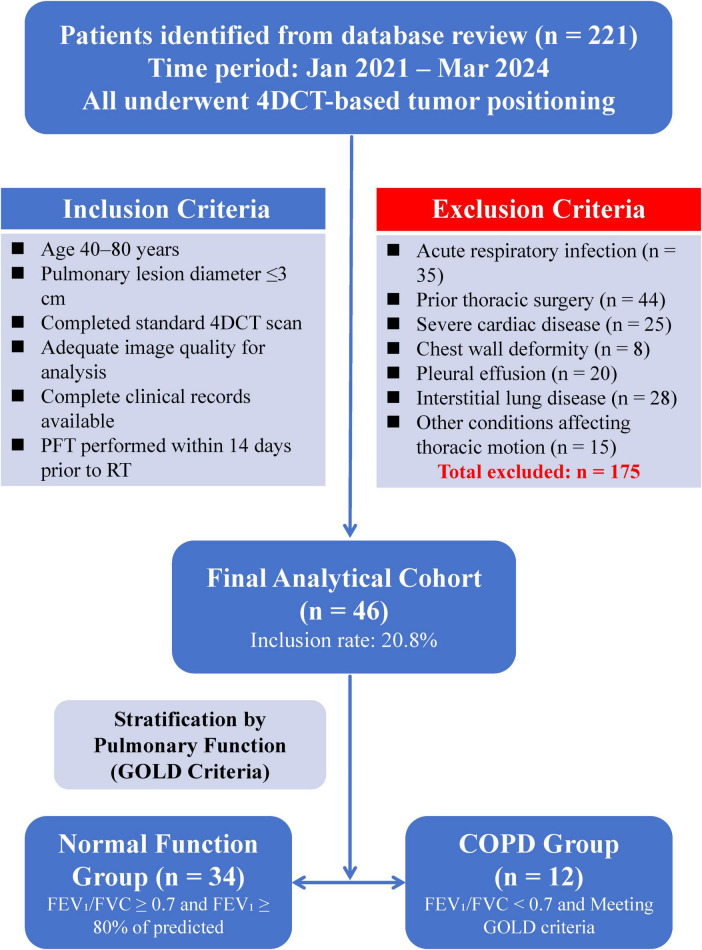
Study flowchart showing patient selection and cohort stratification. Of 221 patients who underwent 4DCT tumor positioning, 175 were excluded based on clinical and imaging criteria. The final analytical cohort (*n* = 46) was divided into preserved lung function (*n* = 34) and COPD (*n* = 12) groups according to pulmonary function test results and GOLD criteria.

Patient selection followed a systematic approach (Figure 1) to identify suitable study subjects. Comprehensive database review initially identified 221 patients who underwent 4D-CT localization scanning in the radiation oncology department during the study period. Inclusion criteria were designed to ensure data quality and analytical validity: patients aged 40–85 years with pulmonary lesions of maximum diameter ≤ 3 cm, completion of standard 4D-CT localization scanning with image quality suitable for strain analysis, complete clinical records including demographic data and symptom assessment, and pulmonary function testing within 2 weeks prior to radiotherapy initiation. Exclusion criteria comprehensively addressed potential confounding factors affecting pulmonary motion analysis: acute respiratory infections within 4 weeks of imaging (*n* = 35), history of thoracic surgery including lobectomy or pneumonectomy (*n* = 44), severe cardiac disease with ejection fraction < 40% or significant valvular disease (*n* = 25), chest wall deformities (*n* = 8), significant pleural effusion > 500 mL (*n* = 20), diaphragmatic paralysis, interstitial lung disease including idiopathic pulmonary fibrosis (*n* = 28), and other conditions affecting lung motion such as massive atelectasis or large mediastinal masses (*n* = 15). Following rigorous screening, 46 patients were included in the final analysis (inclusion rate: 20.8%).

Patients were stratified into two groups based on pulmonary function test results according to established GOLD diagnostic criteria: normal lung function group (*n* = 34; FEV_1_/FVC ≥ 0.7 and FEV_1_ ≥ 80% predicted) and COPD group (*n* = 12; FEV_1_/FVC < 0.7). This classification ensured clear diagnostic separation between groups and aligned with current clinical practice guidelines.

### 2.3 Clinical data collection

Clinical data were retrospectively extracted from electronic medical records through systematic documentation review by two independent reviewers. The comprehensive data collection protocol encompassed demographic information (age, gender, height, weight, body mass index), detailed smoking history including pack-years calculation, and comorbidity assessment. Tumor characteristics were meticulously documented, including histological type determined by biopsy or surgical pathology, precise lesion size measurements from CT imaging using standardized techniques, and anatomical location classification by pulmonary lobe. The maximum diameter was measured in the axial plane using electronic calipers on the treatment planning CT scan. Clinical symptom assessment included systematic evaluation of cough (defined as persistent cough for > 8 weeks), sputum production (daily expectoration), and dyspnea severity using the modified Medical Research Council (mMRC) dyspnea scale. The mMRC scale ranges from 0 (dyspnea only with strenuous exercise) to 4 (too dyspneic to leave house or breathless when dressing/undressing), providing standardized functional impairment evaluation.

All patients underwent standard pulmonary function testing prior to radiotherapy according to American Thoracic Society guidelines using calibrated spirometry equipment (MasterScope, Jaeger, Germany). Key spirometric parameters were documented with both absolute values and percent predicted based on age, height, gender, and ethnicity: forced vital capacity (FVC), forced expiratory volume in 1 s (FEV_1_), FEV_1_/FVC ratio, peak expiratory flow (PEF), and mid-expiratory flow at 50% vital capacity (MEF_50_). All measurements were performed by certified respiratory technicians, with the best of three acceptable maneuvers recorded.

### 2.4 4 D-CT scanning protocol

All patients underwent standardized 4D-CT localization scanning according to established radiation oncology protocols ensuring consistency and reproducibility. Imaging was performed using a Siemens SOMATOM Emotion 16-slice CT scanner with optimized parameters balancing image quality and radiation dose considerations.

Technical parameters included: 120 kV tube voltage, 60–100 mA tube current (adjusted for patient body habitus using automatic exposure control), 0.5-s rotation time, 16 × 0.75 mm collimation, and helical acquisition with 0.9 pitch. Patients were positioned supine with arms elevated above the head using standard radiotherapy immobilization devices to ensure reproducible positioning.

Image reconstruction utilized standardized parameters: 2.5 mm slice thickness, 2.5 mm slice spacing, standard reconstruction algorithm (B31f), and 350–400 mm field of view adjusted for patient body habitus. Respiratory-gated acquisition was performed using the Real-time Position Management (RPM) system (Varian Medical Systems, Palo Alto, CA), which tracks external respiratory motion using an infrared camera and reflective markers placed on the patient’s chest.

The 4D-CT acquisition protocol captured the complete respiratory cycle, with images sorted into 10 respiratory phases (0–90% of the breathing cycle) using phase-based binning. For strain analysis, end-inspiration (EI, 0% phase) and end-expiration (EE, 50% phase) images were selected based on maximum and minimum lung volumes, respectively.

### 2.5 4 D-CT image processing and strain analysis

#### 2.5.1 Data preprocessing and lung segmentation

The 4D-CT image datasets underwent standardized preprocessing to ensure analytical accuracy and reproducibility. Raw DICOM sequences were first validated for completeness, verifying the continuity and consistency across all respiratory phases. To balance computational efficiency with image quality preservation, adaptive resampling was performed with a resampling factor of 0.5, while maintaining spatial resolution of critical anatomical structures. The SimpleITK library was employed for DICOM series reading, ensuring accurate preservation of image orientation, spacing, and origin information throughout the processing pipeline.

Lung parenchyma segmentation was achieved through an adaptive threshold-based algorithm integrated with morphological operations. The segmentation workflow comprised four sequential steps. Initial threshold segmentation employed −400 HU as the primary threshold value, which was empirically validated to effectively differentiate lung parenchyma from surrounding soft tissues. Connected component analysis using 3D labeling algorithms identified independent anatomical structures, excluding noise regions smaller than 1,000 pixels and artifacts located near image boundaries. Morphological optimization was performed using binary closing operations with a 5-pixel radius structuring element to fill small holes and gaps caused by vascular shadows within the lung parenchyma. Finally, n-dimensional binary hole filling algorithms processed internal airway and vascular structures to generate complete lung masks. Given that lesions in our cohort were small (≤ 3,cm, representing < 5% of total lung volume based on average lung capacity of 3,000–4,000 mL), their biomechanical impact was considered negligible while providing a more comprehensive evaluation of global lung mechanics.

#### 2.5.2 Deformable image registration

Respiratory motion registration employed a hierarchical approach combining centroid-based rigid registration for initial estimation followed by refinement procedures. The end-expiratory phase (phase 0) was selected as the registration reference due to its superior image quality and minimal motion artifacts. Centroid registration calculated the center of mass coordinates for each phase’s lung mask, estimating initial translation parameters through centroid differences. Fine registration was subsequently performed using mutual information metrics to compensate for spatial displacements caused by respiratory motion, building upon the centroid registration foundation.

Dense optical flow fields between respiratory phases were computed using the Farneback algorithm with optimized parameters. The optical flow calculation employed a pyramidal strategy, initially computing coarse displacement on downsampled images before upsampling to original resolution for refinement. To reduce computational burden, two-fold downsampling was applied during initial calculations. The algorithm parameters were set as follows: pyramid scale 0.5, pyramid levels 3, window size 15, iterations 3, polynomial neighborhood 5, polynomial sigma 1.2, and flags 0. Registration accuracy was rigorously evaluated using target registration error (TRE) measurements at anatomical landmarks including vessel bifurcations and fissures. Stringent acceptance criteria required TRE values < 2 mm across all lung regions, with studies failing this criterion excluded from analysis.

#### 2.5.3 Strain parameter calculation

Dense displacement fields were calculated using the advanced Farneback optical flow algorithm, a robust method for tracking tissue motion that has been extensively validated in medical imaging applications. This algorithm provides pixel-level displacement vectors throughout the lung parenchyma, enabling comprehensive motion characterization. Strain tensors were calculated using fundamental biomechanical relationships based on the spatial derivatives of displacement fields: ε_ij_ = 1/2(∂u_i_/∂x_j_ + ∂u_j_/∂x_i_), where ε_ij_ represents strain tensor components and u_i_ represents displacement components in the i-th direction. Principal strain values were obtained through eigenvalue decomposition of strain tensors, enabling extraction of physiologically relevant deformation measurements independent of coordinate system orientation. Three key strain parameters were systematically extracted for each patient:

*Maximum Principal Strain (PSmax)*: Represents the maximum tissue deformation capability across all lung regions, reflecting peak mechanical response during respiratory motion. This parameter indicates the lung’s capacity for elastic deformation and is expressed in millimeters.

*Mean Principal Strain (PSmean):* Quantifies the average deformation level across analyzed tissue regions, providing overall assessment of tissue compliance and mechanical homogeneity. This parameter reflects global lung mechanical behavior and is expressed in millimeters.

*Maximum Displacement Velocity (Speedmax)*: Calculated as displacement magnitude divided by the temporal interval between respiratory phases, quantifying dynamic respiratory motion aspects. This parameter captures the velocity characteristics of lung tissue movement and is expressed in millimeters per second.

**FIGURE 2:**
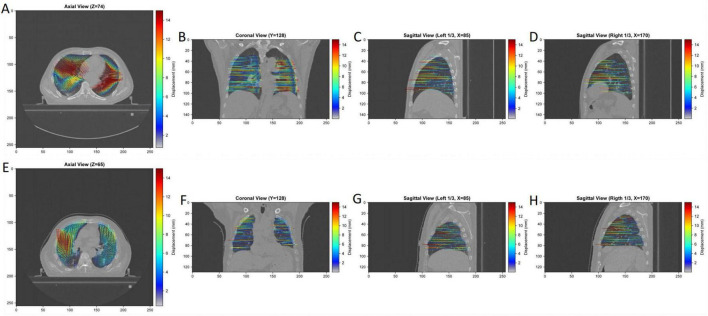
Representative strain analysis vector maps. **(A–D)** 72-year-old male with BMI of 29.04 kg/m^2^, no smoking history, no significant cough or sputum symptoms, dyspnea grade 0, FEV_1_/FVC ratio of 76.44%, MEF_50_ of 2.67 L/s, PSmax of 17.80 mm, PSmean of 2.20 mm, and Speedmax of 1333.37 mm/s. **(E–H)** 78-year-old male with BMI of 24.6 kg/m^2^, 40-year smoking history, presenting with cough and sputum symptoms, dyspnea grade 2, FEV_1_/FVC ratio of 42.84%, MEF_50_ of 0.23 L/s, PSmax of 17.16 mm, PSmean of 2.36 mm, and Speedmax of 661.09 mm/s.

Figure 2 demonstrates representative 4D-CT images and principal strain analysis results from both normal and COPD patients, illustrating the clear differences in strain patterns between groups. The comprehensive processing workflow code has been made available at https://github.com/wmshxf/4dct-strain-analysis for research reproducibility.

### 2.6 End-inspiration CT quantitative analysis

**FIGURE 3:**
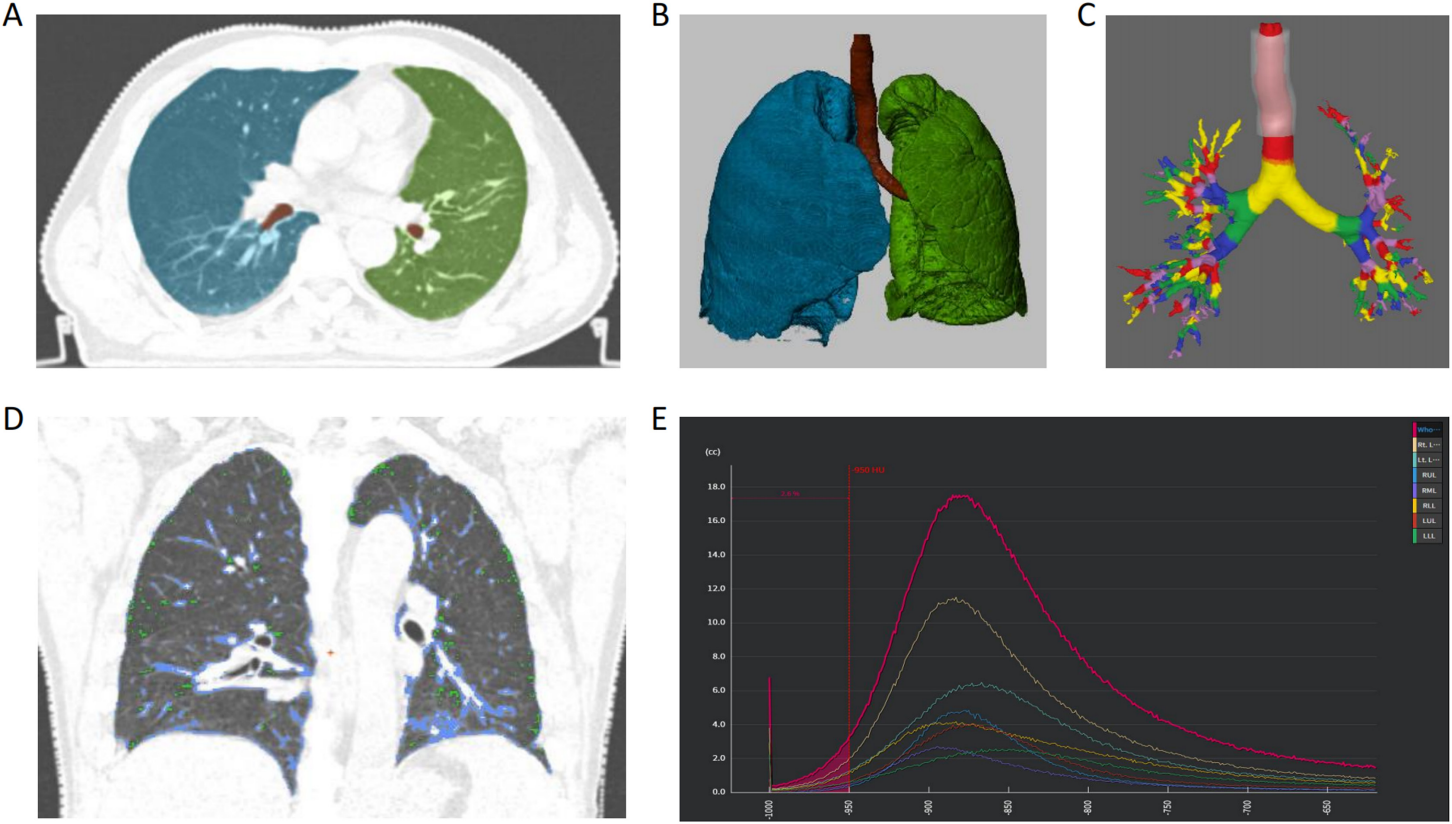
Aview^®^ system CT quantitative analysis schematic diagram. Automatic identification of lung lobes and airways **(A)**, generating volume rendering (VR) of left and right lung lobes and bronchial tree **(B)**, different colors representing quantified airway classification **(C)**; through threshold segmentation **(D)**, green areas represent low attenuation areas (LAA, < -950 HU), blue areas represent high attenuation areas (HAA, –600 to –250 HU); density histograms of whole lung and individual lung lobes intuitively display the proportion of LAA regions (E).

End-inspiration CT images were selected for quantitative analysis based on respiratory gating displays showing maximum lung expansion. Quantitative lung CT analysis was performed using the validated Aview^®^ system (Coreline Soft Inc., Seoul, South Korea), which provides automated measurement of established emphysema and airway parameters (Figure 3). Lung parenchymal characteristics were determined through standardized histogram threshold methods: pixel index PI-15 (percentage of lung voxels with attenuation values ≤ -950 HU at end-inspiration), low attenuation area percentage (LAA%, percentage of lung area with attenuation ≤ −950 HU), and mean lung density (MLD, average attenuation of lung parenchyma in Hounsfield units). These parameters quantify emphysema extent and severity according to established radiological criteria. Airway structural characteristics were quantified using the square root of wall area for airways with 10 mm internal perimeter (Pi10), a standardized measure of airway wall thickness that correlates with small airway disease severity. Airway measurements were performed on airways perpendicular to the imaging plane to minimize partial volume effects. All quantitative measurements were performed by a single experienced operator (B.C.) blinded to clinical data and strain analysis results to minimize bias. Quality control included visual verification of automated segmentations and manual correction when necessary.

### 2.7 Statistical analysis

Statistical analysis was performed using SPSS version 26.0 (IBM Corp., Armonk, NY) and R version 4.2.0 (R Foundation for Statistical Computing, Vienna, Austria). Continuous variables were assessed for normality using the Shapiro-Wilk test and Q-Q plots, and group comparisons were conducted using independent samples *t*-tests or Mann-Whitney U tests as appropriate; categorical variables were analyzed using chi-square or Fisher’s exact tests. Spearman’s rank correlation coefficient was used to evaluate associations between Speedmax and physiological parameters. Correlation coefficients were interpreted as weak (0.1–0.3), moderate (0.3–0.5), or strong (> 0.5). Receiver operating characteristic (ROC) curve analysis was employed to assess the diagnostic performance of Speedmax for COPD detection, with area under the curve (AUC), optimal cutoff values (via Youden’s index), sensitivity, specificity, and corresponding 95% confidence intervals reported; DeLong’s test was used for AUC comparisons. To account for potential confounding by lesion size, multivariate logistic regression models were fitted with COPD status as the outcome, and internal validation was performed using bootstrap resampling (1,000 replicates) to compute bias-corrected AUC, sensitivity, and specificity. All tests were two-tailed, and statistical significance was defined as *P* < 0.05. Missing data were handled using complete case analysis (listwise deletion) for each specific analysis. This conservative approach was chosen to avoid introducing imputation-related bias given our small sample size.

## 3 Results

### 3.1 Baseline clinical characteristics

The study population included 34 patients with normal lung function and 12 COPD patients (Table 1). COPD patients demonstrated older age trend (72.08 ± 6.08 vs. 67.29 ± 9.52 years, *P* = 0.055, Cohen’s *d* = 0.545) and significantly lower body weight (60.21 ± 7.90 vs. 66.52 ± 10.62 kg, *P* = 0.044, *d* = 0.634) compared to the normal function group. Both groups exhibited male predominance (COPD: 91.7%; Normal: 79.4%) with comparable smoking histories. Respiratory symptoms were markedly more prevalent among COPD patients, with significantly higher rates of cough (83.3% vs. 41.2%, *P* = 0.029), sputum production (83.3% vs. 35.3%, *P* = 0.011), and dyspnea (mMRC ≥ 2: 75.0% vs. 0.0%, *P* < 0.001).

**TABLE 1:** Baseline characteristics of study participants stratified by lung function status.

Clinical parameter	Normal function group (*n* = 34)	COPD group (*n* = 12)	*P*-value	Cohen’s *d*
Age (years)	67.29 ± 9.52	72.08 ± 6.08	0.055	0.545
Male gender, n(%)	27(79.4)	11(91.7)	0.603	–
Height (m)	1.70 ± 0.06	1.66 ± 0.06	0.094	0.576
Weight (kg)	66.52 ± 10.62	60.21 ± 7.90	0.044	0.634
BMI (kg/m^2^)	23.16 ± 3.29	21.86 ± 2.75	0.203	0.413
Smoking history, n(%)	15(44.1)	6(50.0)	0.988	–
Pack-years	15.15 ± 18.11	19.58 ± 21.04	0.524	0.235
**Tumor characteristics**				
Primary lung cancer, n(%)	29(85.3)	11(91.7)	1.000	–
Metastatic lesion, n(%)	5(14.7)	1(8.3)	1.000	–
Lesion size (cm)	1.94 ± 0.31	2.40 ± 0.23	< 0.001	1.553
Upper lobe lesion, n(%)	17(50.0)	6(50.0)	1.000	–
Lower lobe lesion, n(%)	17(50.0)	6(50.0)	1.000	–
**Symptoms**				
Cough, n(%)	14(41.2)	10(83.3)	0.029	–
Sputum production, n(%)	12(35.3)	10(83.3)	0.011	–
Dyspnea (mMRC ≥ 2), n(%)	0(0.0)	9(75.0)	< 0.001	–
**PFT**				
FVC (L)	2.88 ± 0.73	1.92 ± 0.55	< 0.001	1.389
FVC% predicted	3.61 ± 0.60	3.35 ± 0.44	0.132	0.453
FEV_1_ (L)	2.37 ± 0.63	1.13 ± 0.40	< 0.001	2.127
FEV_1_% predicted	2.84 ± 0.45	2.61 ± 0.37	0.101	0.522
FEV_1_/FVC (%)	82.31 ± 6.41	58.44 ± 8.78	< 0.001	3.373
PEF (L/s)	5.61 ± 1.80	3.22 ± 1.07	< 0.001	1.453
MEF_50_ (L/s)	2.96 ± 1.17	0.68 ± 0.35	< 0.001	2.210

Values are presented as mean ± SD or n(%). *P*-values from independent *t*-tests, Mann-Whitney U tests, chi-square tests, or Fisher’s exact tests as appropriate. BMI, body mass index; FVC, forced vital capacity; FEV_1_, forced expiratory volume in one second; PEF, peak expiratory flow; MEF_50_, mid-expiratory flow at 50% vital capacity; mMRC, modified Medical Research Council dyspnea scale.

Primary lung cancer comprised the majority of cases in both groups (COPD: 91.7%; Normal: 85.3%). Lesion size differed significantly between groups (COPD: 2.40 ± 0.23 cm vs. Normal: 1.94 ± 0.31 cm, *P* < 0.001, *d* = 1.553), with similar anatomical distribution between upper and lower lobes across groups (50% each).

Pulmonary function testing revealed significant impairment across all measured parameters in COPD patients, validating the diagnostic stratification: FEV_1_/FVC (58.44 ± 8.78% vs. 82.31 ± 6.41%, *P* < 0.001, *d* = 3.373), FEV_1_ (1.13 ± 0.40 vs. 2.37 ± 0.63 L, *P* < 0.001, *d* = 2.127), PEF (3.22 ± 1.07 vs. 5.61 ± 1.80 L/s, *P* < 0.001, *d* = 1.453), and MEF_50_ (0.68 ± 0.35 vs. 2.96 ± 1.17 L/s, *P* < 0.001, *d* = 2.210). These baseline characteristics confirmed appropriate patient classification and reflected the expected clinical profiles of COPD versus normal lung function populations among lung cancer patients undergoing radiotherapy planning.

### 3.2 4 D-CT strain parameters and statistical power

#### 3.2.1 Between-group comparisons

Strain parameter analysis demonstrated significant biomechanical differences between groups (Table 2). Speedmax exhibited the most substantial reduction in COPD patients, achieving statistical significance with a large effect size (763.22 ± 85.22 vs. 1074.19 ± 319.68 mm/s, *P* < 0.001, Cohen’s *d* = 1.114). This finding reflects impaired respiratory dynamics consistent with the pathophysiological changes of elastic fiber degradation and increased airway resistance in COPD.

**TABLE 2:** 4D-CT strain analysis parameters: comparison between normal lung function and COPD groups.

Strain parameter	Normal function group	COPD group	*P*-value	Cohen’s *d*
PSmax (mm)	26.32 ± 8.71	22.70 ± 6.58	0.148	0.441
PSmean (mm)	2.51 ± 0.88	2.14 ± 0.54	0.097	0.465
Speedmax (mm/s)	1074.19 ± 319.68	763.22 ± 85.22	< 0.001	1.114

Values are presented as mean ± SD. *P*-values from independent *t*-tests. PSmax, Maximum Principal Strain; PSmean, Mean Principal Strain; Speedmax, Maximum Displacement Velocity. Statistical power calculated for Speedmax using observed effect size (Cohen’s *d* = 1.114), sample sizes (Normal: *n* = 34, COPD: *n* = 12), and α = 0.05.

Both PSmax (22.70 ± 6.58 vs. 26.32 ± 8.71 mm, *P* = 0.148, *d* = 0.441) and PSmean (2.14 ± 0.54 vs. 2.51 ± 0.88 mm, *P* = 0.097, *d* = 0.465) showed trends toward reduction in COPD patients, with medium effect sizes approaching statistical significance. These parameters indicated reduced tissue deformation capability and diminished global tissue compliance in the COPD cohort. The consistent directional changes across all strain parameters provided evidence that COPD fundamentally alters pulmonary biomechanical properties.

#### 3.2.2 *Post hoc* power analysis

Despite the small COPD sample size (*n* = 12), *post hoc* power analysis demonstrated adequate statistical power for detecting the observed difference in Speedmax between groups. With the observed large effect size (Cohen’s *d* = 1.114), sample sizes (Normal: *n* = 34, COPD: *n* = 12), and two-tailed significance level (α = 0.05), the achieved statistical power was 88.1%, exceeding the conventional 80% threshold. This indicates sufficient power to detect clinically meaningful between-group differences despite limited sample size, supporting the validity of our primary findings.

### 3.3 CT quantitative parameters

CT quantitative analysis revealed significant structural differences between groups, validating patient classification and providing complementary evidence to functional strain findings (Table 3). COPD patients demonstrated significantly elevated low attenuation areas (LAA: 7.30 ± 4.18% vs. 3.23 ± 1.43%, *P* = 0.006, *d* = 1.672) and reduced mean lung density (MLD: −889.23 ± 77.02 vs. −767.79 ± 68.58 HU, *P* < 0.001, *d* = 1.717), both indicating emphysematous changes consistent with COPD pathophysiology. PI-15 values were significantly lower in the COPD group (−890.92 ± 42.57 vs. −844.68 ± 50.86 HU, *P* = 0.005, *d* = 0.945), further confirming emphysematous tissue destruction. The concordant findings across LAA, MLD, and PI-15 parameters provided robust evidence of structural lung damage in COPD patients.

**TABLE 3:** End-inspiration CT quantitative analysis parameters comparing normal lung function and COPD groups.

CT parameter	Normal function group	COPD group	*P*-value	Cohen’s *d*
PI-15 (HU)	-844.68 ± 50.86	-890.92 ± 42.57	0.005	0.945
LAA (%)	3.23 ± 1.43	7.30 ± 4.18	0.006	1.672
MLD (HU)	-767.79 ± 68.58	-889.23 ± 77.02	< 0.001	1.717
Pi10 (mm)	2.66 ± 0.53	2.58 ± 0.24	0.520	0.162

Values are presented as mean ± SD. *P*-values from independent *t*-tests or Mann-Whitney U tests as appropriate. PI-15, pixel index at -950 HU threshold; LAA, low attenuation area percentage; MLD, mean lung density; Pi10, square root of wall area for airways with 10 mm internal perimeter.

Conversely, Pi10 measurements showed no significant between-group differences (2.58 ± 0.24 vs. 2.66 ± 0.53 mm, *P* = 0.520, *d* = 0.162), indicating that airway wall thickness abnormalities were not prominent in this cohort. This observation underscores the complementary diagnostic value of 4D-CT strain analysis, which can detect functional biomechanical alterations that may exist independently of quantifiable structural airway changes.

### 3.4 Correlation analysis

**FIGURE 4:**
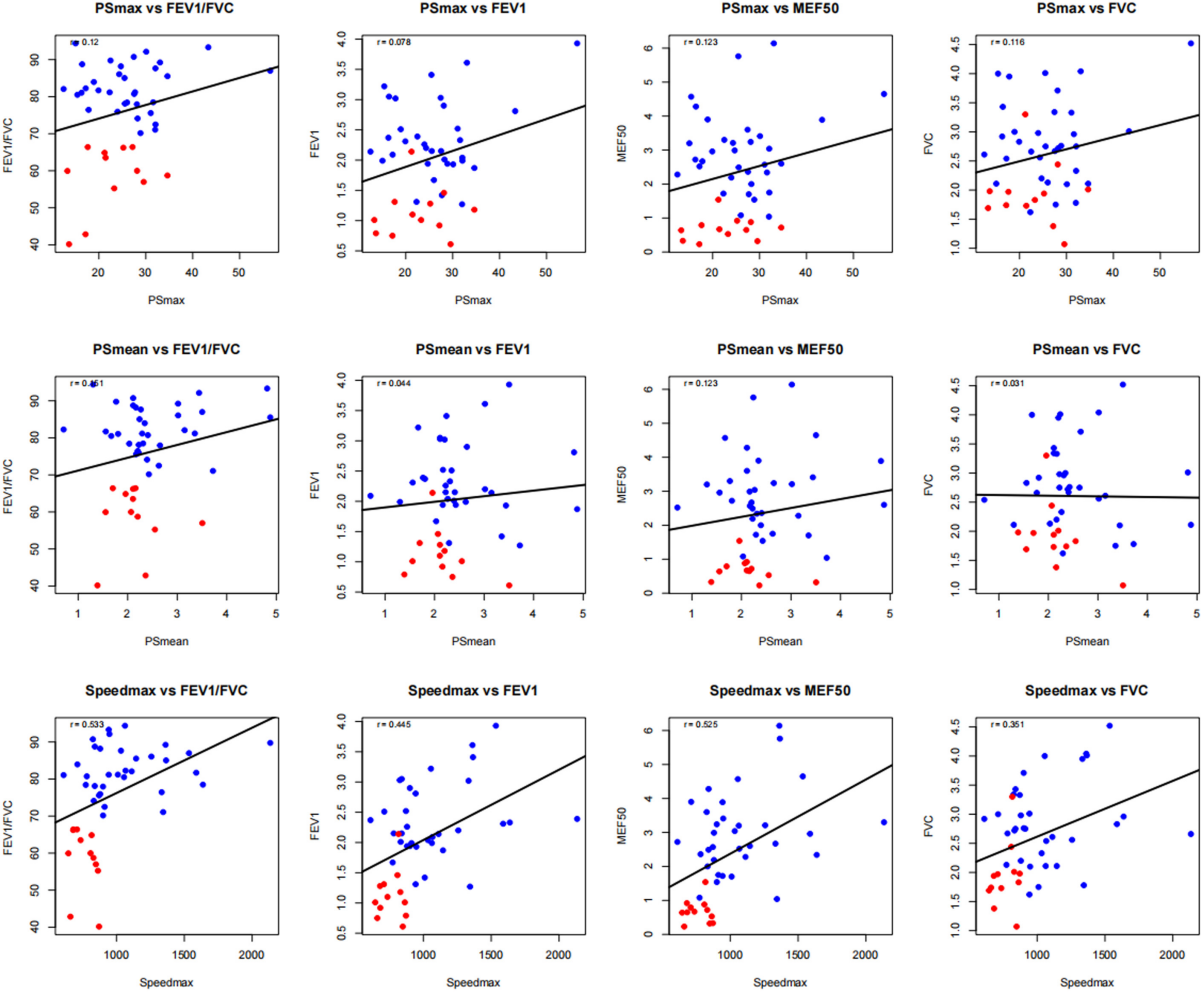
Correlation analysis between 4D-CT strain parameters and spirometric function indices. Scatter plot analysis demonstrating correlations between 4D-CT strain parameters and pulmonary function measurements in 46 patients with small pulmonary lesions. Blue circles represent normal lung function group (*n* = 34), red circles represent COPD group (*n* = 12), solid lines indicate linear regression fit.

Spearman correlation analysis revealed clinically meaningful relationships between strain parameters and pulmonary function measurements (Figure 4 and Table 4). Speedmax demonstrated the strongest correlations across multiple pulmonary function parameters, establishing its biological relevance as a biomechanical marker of respiratory function.

**TABLE 4:** Correlation analysis of strain parameters and pulmonary function indices.

Parameter	FEV_1_/FVC	FEV_1_	MEF_50_	FVC
PSmax	0.120	0.078	0.123	0.116
PSmean	0.151	0.044	0.123	0.031
Speedmax	0.533**	0.445**	0.525**	0.351*

Values represent Spearman correlation coefficients.

*P < 0.05;

**P < 0.01. FEV_1_, forced expiratory volume in 1 s; FVC, forced vital capacity; MEF_50_, mid-expiratory flow at 50% vital capacity.

The correlation between Speedmax and FEV_1_/FVC (*r* = 0.533, *P* < 0.01) was particularly robust, supporting Speedmax as a quantitative marker of airflow obstruction severity. Strong correlations were also observed between Speedmax and FEV_1_ (*r* = 0.445, *P* < 0.01) and MEF_50_ (*r* = 0.525, *P* < 0.01), with the latter being noteworthy given MEF_50_’s sensitivity to small airway dysfunction—a key early feature of COPD pathophysiology. The moderate correlation between Speedmax and FVC (*r* = 0.351, *P* < 0.05) indicated that dynamic motion parameters reflect comprehensive respiratory mechanics beyond isolated airflow limitation. In contrast, PSmax and PSmean showed weaker, non-significant correlations with pulmonary function indices, demonstrating that dynamic motion parameters exhibit superior sensitivity to functional impairment compared to static deformation measures.

### 3.5 Diagnostic performance

#### 3.5.1 ROC analysis and primary diagnostic performance

**FIGURE 5:**
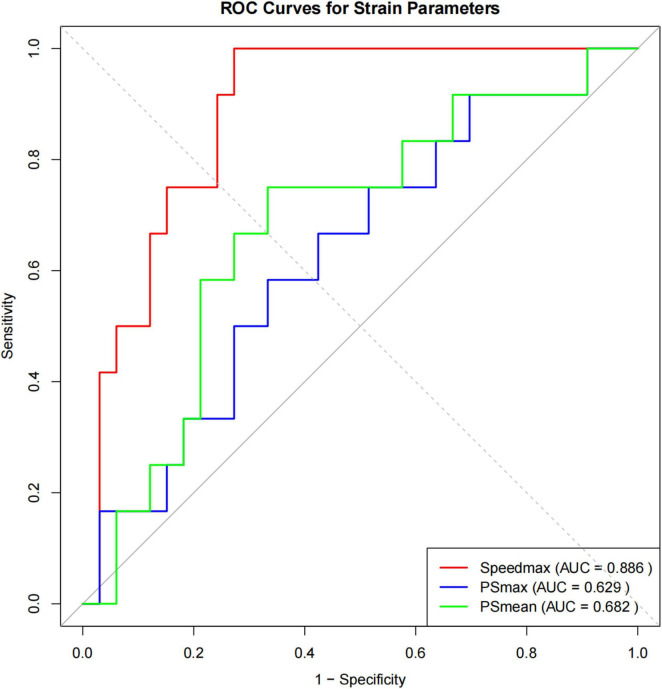
ROC curve analysis evaluating the diagnostic performance of 4D-CT strain parameters. Speedmax demonstrated the best diagnostic performance with optimal sensitivity (100.0%) and specificity (72.7%) at cutoff value of 876.15 mm/s.

ROC curve analysis demonstrated good diagnostic performance of Speedmax for differentiating normal lung function from COPD (Figure 5 and Table 5). Speedmax achieved strong discriminatory ability with an AUC of 0.886 (95% CI: 0.791–0.982, *P* < 0.001), significantly outperforming other strain parameters. At the optimal cutoff value of 876.15 mm/s determined by Youden’s index, Speedmax demonstrated perfect sensitivity (100.0%) and good specificity (72.7%), with a perfect negative predictive value (100.0%). This indicates that all COPD patients had values below the threshold, establishing utility for screening and exclusion of COPD.

**TABLE 5:** Receiver operating characteristic analysis and diagnostic performance of 4D-CT strain parameters for COPD detection.

Parameter	AUC (95% CI)	*P*-value	Optimal cutoff	Sensitivity (%)	Specificity (%)	PPV (%)	NPV (%)
Speedmax	0.886 (0.791–0.982)	< 0.001	876.15	100.0	72.7	57.1	100.0
PSmax	0.629 (0.443–0.814)	0.153	23.63	58.3	66.7	38.9	81.5
PSmean	0.682 (0.502–0.862)	0.043	2.20	75.0	66.7	45.0	88.0

AUC, area under the curve; PPV, positive predictive value; NPV, negative predictive value; CI, confidence interval. Optimal cutoff determined by Youden’s index. DeLong’s test showed Speedmax significantly outperformed PSmax (*P* = 0.002) and PSmean (*P* = 0.031).

PSmean showed statistically significant but modest diagnostic capability (AUC = 0.682, 95% CI: 0.502–0.862, *P* = 0.043) with balanced sensitivity (75.0%) and specificity (66.7%), while PSmax demonstrated the weakest performance (AUC = 0.629, 95% CI: 0.443–0.814, *P* = 0.153), failing to achieve statistical significance. DeLong’s test confirmed significantly superior performance of Speedmax compared to both PSmax (*P* = 0.002) and PSmean (*P* = 0.031).

#### 3.5.2 Bootstrap internal validation results

Internal validation using 1,000 bootstrap replicates assessed model stability and potential overfitting (Table 6). The analysis revealed remarkable consistency between original and bootstrap-estimated performance, with the original AUC of 0.888 closely matched by the bootstrap mean AUC of 0.887. The optimism bias was minimal at −0.001, yielding an optimism-corrected AUC of 0.889 (95% CI: 0.776–0.971). This negligible optimism bias indicates minimal overfitting, with the optimism-corrected AUC nearly identical to the original estimate. Bootstrap mean sensitivity reached 97.1% (95% CI: 77.8–100.0%) and specificity 78.2% (95% CI: 58.6–96.6%), demonstrating robustness of the diagnostic model despite small sample size. These findings confirm that the diagnostic performance observed in our cohort is not attributable to overfitting and likely generalizable within similar populations.

**TABLE 6:** Bootstrap internal validation results for Speedmax diagnostic performance.

Performance metric	Original value	Bootstrap mean (1,000 iterations)	Optimism (bias)	Optimism-corrected value	95% CI
AUC	0.888	0.887	−0.001	0.889	0.776–0.971
Sensitivity (%)	100.0	97.1	−2.9	100.0*	77.8–100.0
Specificity (%)	72.4	78.2	+ 5.8	66.6	58.6–96.6

*Optimism-corrected sensitivity capped at 100% for clinical interpretation. CI, confidence interval; AUC, area under the curve. Bootstrap resampling with 1,000 iterations used to assess model stability and overfitting. Bias-corrected and accelerated (BCa) 95% confidence intervals reported.

### 3.6 Multivariate analysis and tumor size adjustment

#### 3.6.1 Multivariate logistic regression

To address the significant between-group difference in lesion size and evaluate whether Speedmax remains an independent predictor after adjusting for this potential confounder, multivariate logistic regression was performed (Table 7). Model 1 (univariate) included Speedmax alone as predictor, yielding a coefficient of −0.01211 (SE: 0.0045) with an odds ratio of 0.988 (95% CI: 0.980–0.996, *P* < 0.05), AUC of 0.888, and log loss of 0.3946. Model 2 (multivariate) incorporated both Speedmax and lesion size as predictors. In this multivariate model, Speedmax demonstrated a coefficient of −0.01070 (SE: 0.0048) with an odds ratio of 0.989 (95% CI: 0.981–0.998, *P* < 0.05), while lesion size showed a coefficient of 1.216 (SE: 0.598) with an odds ratio of 3.374 (95% CI: 1.045–10.889, *P* < 0.05). The multivariate model achieved an improved AUC of 0.931, representing an increase of 0.043 compared to the univariate model.

**TABLE 7:** Multivariate logistic regression analysis results.

Model	Predictors	Coefficient	SE	Odds ratio	95% CI	*P-*value	AUC	AUC improvement
Model 1	Speedmax	−0.01211	0.0045	0.988	0.980-0.996	< 0.05	0.888	–
Model 2	Speedmax	−0.01070	0.0048	0.989	0.981–0.998	< 0.05	0.931	+ 0.043
	Lesion size	1.216	0.598	3.374	1.045–10.889	< 0.05		

Model 1: Univariate logistic regression with Speedmax as single predictor. Model 2: Multivariate logistic regression with both Speedmax and lesion size as predictors. Outcome variable: COPD status (binary). SE, standard error; CI, confidence interval; AUC, area under the curve.

The multivariate analysis demonstrates that Speedmax remains a statistically significant independent predictor of COPD status (*P* < 0.05) even after adjusting for tumor size. Both predictors contribute independently to COPD classification, with the multivariate model achieving improved discrimination (AUC = 0.931) compared to the univariate model (AUC = 0.888). This confirms that the observed Speedmax reduction in COPD patients is not solely attributable to differences in tumor size.

#### 3.6.2 Tumor size-matched subgroup analysis

Sensitivity analysis using propensity score matching created a tumor size-matched subgroup to further validate findings (Table 8). Among the 12 COPD patients, 11 were successfully matched 1:1 with normal function patients based on lesion size (tolerance: ± 0.3 cm). In the matched subgroup, both groups showed comparable mean lesion sizes (COPD: 2.38 ± 0.24 cm vs. Normal: 2.35 ± 0.28 cm, *P* = 0.742, Cohen’s *d* = 0.108), confirming negligible between-group difference. Despite this elimination of lesion size differences, Speedmax remained significantly reduced in COPD patients (757.12 ± 87.33 vs. 962.45 ± 251.83 mm/s, *P* = 0.031) with a substantial effect size (Cohen’s *d* = 0.943).

**TABLE 8:** Sensitivity analysis: tumor size-matched subgroup comparison.

Parameter	Matched COPD group (*n* = 11)	Matched Normal group (*n* = 11)	*P-*value	Cohen’s d
Lesion size (cm)	2.38 ± 0.24	2.35 ± 0.28	0.742	0.108
Speedmax (mm/s)	757.12 ± 87.33	962.45 ± 251.83	0.031	0.943

Values are presented as mean ± SD. Patients matched 1:1 using propensity score matching based on lesion size (tolerance: ± 0.3 cm). *P*-values from paired *t*-tests. This analysis confirms that Speedmax reduction in COPD patients persists even after eliminating lesion size differences.

In the tumor size-matched subgroup, Speedmax remained significantly reduced in COPD patients despite elimination of lesion size differences. The substantial effect size (*d* = 0.943) in this matched analysis provides strong evidence that Speedmax reduction is primarily driven by COPD-related biomechanical changes rather than tumor size confounding.

### 3.7 Physiological parameters and normalized metrics

#### 3.7.1 Correlation with physiological parameters

Analysis of Speedmax correlations with basic physiological parameters revealed important relationships (Table 9). Speedmax demonstrated a weak negative correlation with age (*r* = −0.298, *P* = 0.045), a moderate positive correlation with height (*r* = 0.412, *P* = 0.005), and a moderate positive correlation with FVC (*r* = 0.351, *P* = 0.017). These correlations indicate that Speedmax is influenced by physiological factors, particularly body height and lung volume. Taller individuals and those with larger lung volumes tend to have higher Speedmax values. The weak negative correlation with age suggests modest age-related decline in respiratory motion velocity. These findings highlight important considerations for developing generalizable diagnostic thresholds.

**TABLE 9:** Correlation of Speedmax with physiological parameters and performance of normalized parameters.

Analysis	Parameter	Value	*P*-value	Effect size
**Correlations**				
	Speedmax vs. age	*r* = −0.298	0.045	Weak negative
	Speedmax vs. height	*r* = 0.412	0.005	Moderate positive
	Speedmax vs. FVC	*r* = 0.351	0.017	Moderate positive
**Normalized parameters**				
Speedmax/FVC	COPD	397.32 ± 54.61 s^–1^		*d* = 0.264
	Normal	373.41 ± 102.38 s^–1^	0.410	
	AUC	0.621 (0.436–0.806)	0.183	
Speedmax/height	COPD	459.07 ± 53.92 (mm/s)/m		*d* = 1.083
	Normal	631.88 ± 186.74 (mm/s)/m	< 0.001	
	AUC	0.883 (0.786–0.979)	< 0.001	

Values for normalized parameters presented as mean ± SD. r, Spearman correlation coefficient; FVC, forced vital capacity; AUC, area under the curve; d, Cohen’s d. The Speedmax/Height ratio maintains diagnostic performance comparable to absolute Speedmax, suggesting potential for height normalization in future studies.

#### 3.7.2 Normalized parameters evaluation

To potentially improve generalizability across diverse populations, we evaluated normalized parameters. The Speedmax/FVC ratio showed values of 397.32 ± 54.61 s^–1^ in COPD patients compared to 373.41 ± 102.38 s^–1^ in the normal group (*P* = 0.410, Cohen’s *d* = 0.264), with an AUC of 0.621 (95% CI: 0.436–0.806, *P* = 0.183). In contrast, the Speedmax/Height ratio demonstrated values of 459.07 ± 53.92 (mm/s)/m in COPD patients versus 631.88 ± 186.74 (mm/s)/m in the normal group (*P* < 0.001, Cohen’s *d* = 1.083), achieving an AUC of 0.883 (95% CI: 0.786–0.979, *P* < 0.001).

Unexpectedly, the Speedmax/FVC ratio showed reduced discriminatory ability compared to absolute Speedmax, likely because COPD patients have proportionally reduced both FVC and Speedmax, diminishing the ratio’s sensitivity. The Speedmax/Height ratio maintained strong diagnostic performance (AUC = 0.883) comparable to absolute Speedmax (AUC = 0.886), suggesting potential utility for height normalization in future multi-center studies. However, optimal cutoff values for normalized parameters would require validation in larger, diverse cohorts.

## 4 Discussion

### 4.1 Principal findings

This exploratory study provides preliminary evidence supporting 4D-CT strain analysis, particularly Speedmax, as a potentially valuable diagnostic approach for differentiating normal lung function from COPD in radiotherapy patients with small pulmonary lesions. The key findings establish several important contributions: (1) Speedmax demonstrated statistically significant reduction in COPD patients with a large effect size (Cohen’s *d* = 1.114, *P* < 0.001), confirming substantial biomechanical differences between groups; (2) Speedmax achieved good diagnostic performance (AUC = 0.886, optimism-corrected AUC = 0.889) with perfect sensitivity (100.0%) and good specificity (72.7%); (3) Strong and significant correlations were observed between Speedmax and multiple pulmonary function indices (*r* = 0.351–0.533); (4) Multivariate analysis confirmed Speedmax as an independent predictor after adjusting for tumor size (multivariate AUC = 0.931); and (5) Bootstrap internal validation showed minimal overfitting (optimism = -0.001), supporting model robustness despite small sample size.

### 4.2 Biomechanical foundation and pathophysiological mechanisms

The significant reduction in Speedmax observed in COPD patients aligns with established pathophysiological mechanisms underlying chronic obstructive lung disease. COPD pathogenesis involves progressive alveolar wall destruction and elastic fiber degradation through chronic inflammatory processes, fundamentally altering lung tissue biomechanical properties (19). The large effect size observed for Speedmax reduction (Cohen’s *d* = 1.114) reflects markedly impaired respiratory dynamics, consistent with loss of elastic recoil and increased airway resistance that characterize COPD pathophysiology (20, 21).

Specific biomechanical changes contributing to reduced Speedmax include: (1) degradation of type I collagen and elastic fibers, reducing tissue elasticity and dynamic response capability; (2) alveolar septal thickening and inflammatory cell infiltration, altering tissue mechanical properties; (3) small airway remodeling with increased wall thickness and luminal narrowing, limiting regional ventilation dynamics; and (4) pulmonary vascular changes affecting overall lung tissue mechanical behavior (22).

The inclusion of tumor-containing regions in our analysis reflects a pragmatic approach to biomechanical assessment in clinical radiotherapy populations. Given that lesions in our cohort were small (≤ 3 cm, representing < 5% of total lung volume), their biomechanical impact was considered minimal. However, we acknowledge that even small tumors, particularly peripheral lesions, may locally alter deformation patterns through mechanisms including tissue stiffening, tethering effects on adjacent parenchyma, and disruption of normal elastic fiber architecture. These local effects could potentially introduce bias in regional strain measurements. Our study design did not allow separation of tumor-specific effects from global COPD-related biomechanical changes. Future studies should consider analyses that exclude or mask tumor regions to isolate COPD-specific biomechanical alterations from tumor-related mechanical distortion. Additionally, stratified analyses by tumor location (central vs. peripheral) would help clarify whether lesion position influences strain parameter measurements differently.

### 4.3 Statistical validity: power analysis and internal validation

Despite the small COPD sample size (*n* = 12)—a limitation we fully acknowledge—several factors support the statistical validity of our findings. The *post hoc* power analysis revealed adequate statistical power (88.1%) for detecting the observed Speedmax difference, attributable to the large effect size (Cohen’s *d* = 1.114). This exceeds conventional power thresholds (80%), indicating sufficient statistical strength to detect clinically meaningful differences.

Bootstrap internal validation with 1,000 iterations demonstrated remarkable stability, with minimal optimism bias (−0.001) and optimism-corrected AUC (0.889) nearly identical to the original estimate (0.888). This indicates negligible overfitting despite small sample size. The bootstrap 95% confidence intervals (AUC: 0.776–0.971; Sensitivity: 77.8–100.0%; Specificity: 58.6–96.6%) provide realistic estimates of performance uncertainty.

However, these internal validation results do not substitute for external validation in independent cohorts. Performance estimates from single-center studies with small sample sizes typically demonstrate optimism that may not generalize to broader populations. The proposed cutoff value and diagnostic performance metrics should therefore be considered preliminary and hypothesis-generating rather than definitive clinical thresholds.

### 4.4 Tumor size confounding: multivariate analysis and matched subgroup

The significant between-group difference in lesion size (*P* < 0.001, *d* = 1.553) represented a critical concern requiring rigorous evaluation. Our comprehensive multivariate analysis and sensitivity analyses address this issue through multiple approaches.

Multivariate logistic regression demonstrated that Speedmax remains a statistically significant independent predictor of COPD status (*P* < 0.05, OR = 0.989) after adjusting for tumor size. Importantly, both Speedmax and lesion size contributed independently to the multivariate model (AUC = 0.931), with tumor size also showing significant predictive value (*P* < 0.05, OR = 3.374). This suggests that larger tumors may independently associate with COPD status, possibly reflecting longer smoking exposure or more advanced disease in COPD patients.

The tumor size-matched subgroup analysis provided additional compelling evidence. After propensity score matching eliminated lesion size differences (*P* = 0.742, *d* = 0.108), Speedmax remained significantly reduced in COPD patients (*P* = 0.031, *d* = 0.943). The preservation of large effect size in this matched analysis confirms that Speedmax reduction is primarily driven by COPD-related biomechanical changes rather than tumor size effects.

Together, these analyses demonstrate that while tumor size is a covariate, it does not invalidate the core finding that Speedmax reflects COPD-related biomechanical alterations.

### 4.5 Comparison with previous studies

Our results advance existing literature by demonstrating diagnostic utility in a well-defined radiotherapy population with rigorous methodology. Xu et al. first applied strain measurements to 4D dynamic ventilation CT, establishing theoretical foundations (23). Our study extends this work by demonstrating statistically significant differences with large effect sizes and good diagnostic accuracy in a clinically relevant population.

The strong correlations between Speedmax and pulmonary function indices observed in our study (*r* = 0.351–0.533) compare favorably with previous investigations. Bodduluri et al. demonstrated associations between CT-derived biomechanical indices and patient outcomes in the COPDGene cohort but reported weaker correlations (24). Our superior performance may reflect our focused patient population and advanced strain analysis methodology.

The diagnostic performance achieved by Speedmax (AUC = 0.886, optimism-corrected = 0.889) represents advancement compared to conventional CT emphysema measurements, which typically achieve AUC values of 0.6–0.7 for COPD detection (25). This improvement suggests that dynamic functional assessment provides complementary information to static structural measurements.

### 4.6 Potential clinical implications

#### 4.6.1 Screening and risk stratification

Speedmax’s perfect sensitivity (100.0%) and excellent negative predictive value (100.0%) position it as a potential screening tool for radiotherapy patients, enabling confident exclusion of COPD. This capability addresses a clinical need for rapid functional assessment, given that 40–70% of lung cancer patients have undiagnosed or subclinical COPD (17). Early identification could enable proactive pulmonary optimization.

However, the moderate positive predictive value (57.1%) indicates that positive findings should trigger confirmatory spirometry rather than serving as standalone diagnostic criteria. This positions strain analysis as a screening adjunct that could improve clinical workflows by focusing detailed testing on patients most likely to benefit.

#### 4.6.2 Integration with radiotherapy workflows

The seamless integration of 4D-CT strain analysis with existing radiotherapy planning workflows represents a practical advantage. Since 4D-CT is routinely acquired for motion management, strain analysis can be implemented without additional radiation exposure, patient positioning, or scheduling burden. This eliminates logistical challenges associated with additional functional imaging studies such as perfusion scintigraphy or hyperpolarized gas MRI.

The regional nature of strain analysis could enable identification of functional lung regions for preservation during treatment planning, supporting functional lung avoidance strategies (26). The strong correlation between Speedmax and MEF_50_ (*r* = 0.525) suggests particular relevance for assessing small airway function, which may be important for predicting radiation pneumonitis risk.

### 4.7 Generalizability limitations and need for external validation

#### 4.7.1 Physiological confounders and population-specific cutoffs

Our analysis revealed significant correlations between Speedmax and basic physiological parameters (height: *r* = 0.412; FVC: *r* = 0.351; age: *r* = −0.298), indicating that Speedmax is influenced by body size, lung volume, and age. The proposed cutoff value (876.15 mm/s) does not account for these physiological confounders and is specific to our single-center cohort with particular demographic characteristics (mean age: 68.3 years; predominantly male: 82.6%; mean height: 1.69 m).

The Speedmax/Height ratio maintained diagnostic performance (AUC = 0.883) comparable to absolute Speedmax, suggesting potential utility for height normalization. However, the Speedmax/FVC ratio showed unexpectedly reduced performance (AUC = 0.621), likely because COPD proportionally reduces both parameters. These findings highlight the complexity of developing universally applicable normalized parameters.

Future studies should investigate population-specific or demographic-adjusted cutoff values, potentially using regression models incorporating age, height, gender, and ethnicity. Multi-center validation across diverse populations is essential to establish generalizable diagnostic thresholds.

#### 4.7.2 External validation requirements

The complete lack of external validation represents the most significant limitation of our study. Internal bootstrap validation demonstrates model stability within our cohort but cannot assess generalizability to different institutions, scanner models, imaging protocols, or patient populations. Performance estimates from single-center studies typically overestimate true performance in external validation by 10–20%.

Therefore, the reported cutoff and performance metrics should be considered exploratory and hypothesis-generating rather than ready for clinical implementation. We explicitly and prominently emphasize that external validation in larger, multi-center prospective studies is an absolute prerequisite before any clinical application.

## 5 Conclusion

This exploratory study provides preliminary evidence that 4D-CT strain analysis, particularly the Speedmax parameter, demonstrates significant potential for differentiating normal lung function from COPD in patients with small pulmonary lesions undergoing radiotherapy planning. The combination of statistically significant group differences (*P* < 0.001), large effect sizes (Cohen’s *d* = 1.114), good diagnostic performance (AUC = 0.889 after optimism correction), and adequate statistical power (88.1%) supports the biological plausibility and clinical relevance of this approach. Multivariate analysis and tumor size-matched subgroup analysis confirmed the independent predictive value of Speedmax after adjusting for tumor size, addressing a critical potential confounding factor. Bootstrap internal validation showed minimal optimism bias (−0.001), confirming model robustness and indicating that findings are not attributable to overfitting despite small sample size. The strong correlations observed between Speedmax and multiple pulmonary function indices, particularly MEF_50_ (*r* = 0.525), suggest that strain analysis captures fundamental aspects of respiratory mechanics closely linked to functional impairment.

However, several critical limitations must be acknowledged. The small COPD sample size (*n* = 12), single-center design, and lack of external validation substantially limit the generalizability of our findings. The proposed cutoff value (876.15 mm/s) is specific to this cohort and does not account for important physiological confounders such as age, height, and lung volume, as evidenced by moderate correlations between Speedmax and these parameters. While our multivariate analysis controlled for tumor size, broader validation accounting for diverse demographic and clinical characteristics is essential. Furthermore, the inclusion of tumor-containing regions in strain calculations may introduce regional bias, and future studies should evaluate the impact of tumor masking on diagnostic performance.

Most importantly, until external validation is completed in adequately powered, multi-center prospective studies, these findings should be interpreted as hypothesis-generating rather than ready for clinical implementation. The current study establishes diagnostic feasibility within a single-center radiotherapy population but does not demonstrate integration into treatment planning systems or impact on clinical outcomes.

While this study does not demonstrate treatment planning integration, it establishes the diagnostic potential of 4D-CT strain analysis in the radiotherapy population. The combination of good diagnostic performance, practical implementation advantages (no additional radiation exposure, integration with existing 4D-CT workflows), and strong biological plausibility positions strain analysis as a promising avenue for future research in functional lung imaging. With appropriate validation and refinement, this approach may eventually contribute to personalized radiotherapy planning and improved patient outcomes, but substantial additional investigation is required before clinical adoption.

## Data Availability

The original contributions presented in this study are included in this article/supplementary material, further inquiries can be directed to the corresponding author.
